# Role of transcranial Doppler in assessment of cerebral blood flow in full term neonates with extreme unconjugated hyperbilirubinemia

**DOI:** 10.1007/s40477-022-00704-0

**Published:** 2022-08-15

**Authors:** Sara Mahmoud Kamel, Reem Mahmoud Badr-Eldin, Mahmoud Mostafa Arafat, Rania H. Hashem

**Affiliations:** 1 Diagnostic and Intervention Radiology Department, Cairo University Hospitals, Kasr Al-Ainy, El-Manial, Cairo, 11956 Egypt; 2 Pediatric Department, Abu El Reesh Hospital, Kasr Al-Ainy, Cairo, Egypt

**Keywords:** Unconjugated hyperbilirubinemia, Transcranial Doppler, Cerebral blood flow, Extreme hyperbilirubinemia, BIND, Jaundice, Full term

## Abstract

**Purpose:**

To evaluate the difference in cerebral blood flow in neonates with and without extreme unconjugated hyperbilirubinemia.

**Methods:**

Transcranial Doppler parameters of 26 full term newborns with extreme unconjugated hyperbilirubinemia (UCH) were compared to 13 postnatal age and sex matched normal healthy neonates serving as controls. Resistance index (RI), pulsatility index (PI) and peak systolic velocity (PSV) were measured in the middle cerebral, internal carotid and posterior cerebral arteries on both sides by transcranial color Doppler ultrasound.

**Results:**

An increase in cerebral blood flow (decreased RI, PI and increased PSV) was observed in the extreme unconjugated hyperbilirubinemia (UCH) group. There was positive correlation between total serum bilirubin level and peak systolic velocity and vice versa with resistivity and pulsatility indices. Eight neonates developed clinical features of acute bilirubin encephalopathy and showed significantly increased peak systolic velocity in the right middle cerebral artery compared to those with normal outcome. Resistivity index and pulsatility index were lower in patients managed by exchange transfusion compared to those managed with phototherapy.

**Conclusion:**

An increase in cerebral blood flow was observed in neonates with UCH compared to those without hyperbilirubinemia. By assessing the cerebral blood flow velocity, resistivity index (RI), and pulsatility index (PI) of particular intracranial arteries, the transcranial Doppler can identify the at-risk neonates, for development of neurological affliction in extreme unconjugated hyperbilirubinemia.

## Introduction

Unconjugated hyperbilirubinemia (UCH) remains one of the most common clinical phenomena in newborns. Approximately 60% of term and 80% of preterm neonates develop UCH in the first week of life [1].

The degree of hyperbilirubinemia varies depending on the etiology. Severe neonatal hyperbilirubinemia might cause long-term neurocognitive and other neurological sequelae, such as non-syndromic auditory neuropathy, deafness, and learning disabilities. [2].The exact level of bilirubin, likely to cause bilirubin-induced neurological dysfunction (BIND) in any individual baby, is difficult to predict, and there is tremendous variation in susceptibility toward bilirubin encephalopathy among newborns for a variety of unexplained reasons [3].

Bilirubin molecule mediates oxidative stress and cerebral damage at higher serum concentrations [4]. The various factors found to be responsible for bilirubin-mediated neurotoxicity include the release of pro-inflammatory cytokines from astrocytes and microglia [5, 6], disruption of glutathione redox status [7], increased expression of neuronal nitric oxide synthase (nNOS) and production of nitric oxide(NO), cyclic guanosine 30,50-monophosphate (cGMP) and reactive oxygen species (ROS) [8].

Since all of these factors have the potential to alter cerebral blood flow (CBF), it was hypothesized that there may be some difference in cerebral blood flow velocity (CBFV) in otherwise healthy neonates with hyperbilirubinemia compared with those without any icterus. Unfortunately, there is no easily available, bedside tool which can depict cerebral dysfunction in neonatal UCH. Magnetic resonance imaging (MRI) of the brain may be an option to pick up neurological abnormality early, but it is expensive and not easily available in all centers of a developing country [9].

Transcranial Doppler (TCD) is a non-invasive, low-cost, and repeatable diagnostic and monitoring tool that can be used in real time bedside examination of cerebral blood circulation, in critically ill patients [10]. It is the only non-invasive examination that enables a valid real-time evaluation of cerebral blood flow patterns, supplementing anatomical information gained from other neuroimaging modalities [11].

## Methods

### Study population

This prospective observational case control study was conducted over a period of 5 months, including two groups of full term neaonates. The first group was 26 full term neonates with extreme unconjugated hyperbilirubinemia, defined as total serum bilirubin level equal to or more than 20 mg/dl, admitted to the neonatal intensive care unit. Both sexes were included, with the age range being from 2 to 7 days old.

The other group recruited was 13 jaundice-free full term neonates as a control group, admitted at the neonatal surgical intensive care unit, for causes other than jaundice.

A verbal consent was obtained from the legal guardians of patients before enrollment in the study.

We excluded preterm neonates,patients with total serum bilirubin level less than 20 mg/dl. We also excluded patients with factors that may have direct or indirect impact on the cerebral blood flow velocity as perinatal asphyxia, shock, sepsis, anemia, systemic and metabolic disorders as well as hypo/hyperthermia.

### Clinical examination

- Full clinical history was taken with emphasis on the mode of delivery, age, sex, diagnosis, neurological manifestation/BIND score and the plan of management.
- The BIND score (Bilirubin-Induced Neurologic Dysfunction) is used to assess the degree of severity of neonatal jaundice and associated acute bilirubin encephalopathy. It depends on assessment of the mental status, muscle tone and the cry pattern.

### Laboratory investigations

- Laboratory data were obtained from the files with emphasis on total serum bilirubin and serum direct bilirubin levels.

### Imaging

All sonographic examinations were performed bed side at the neonatal intensive care unit, by one investigator using CANON XARIO-100 ultrasound device that is equipped by 2–3 MHz phased array probe.

**Fig. 1:**
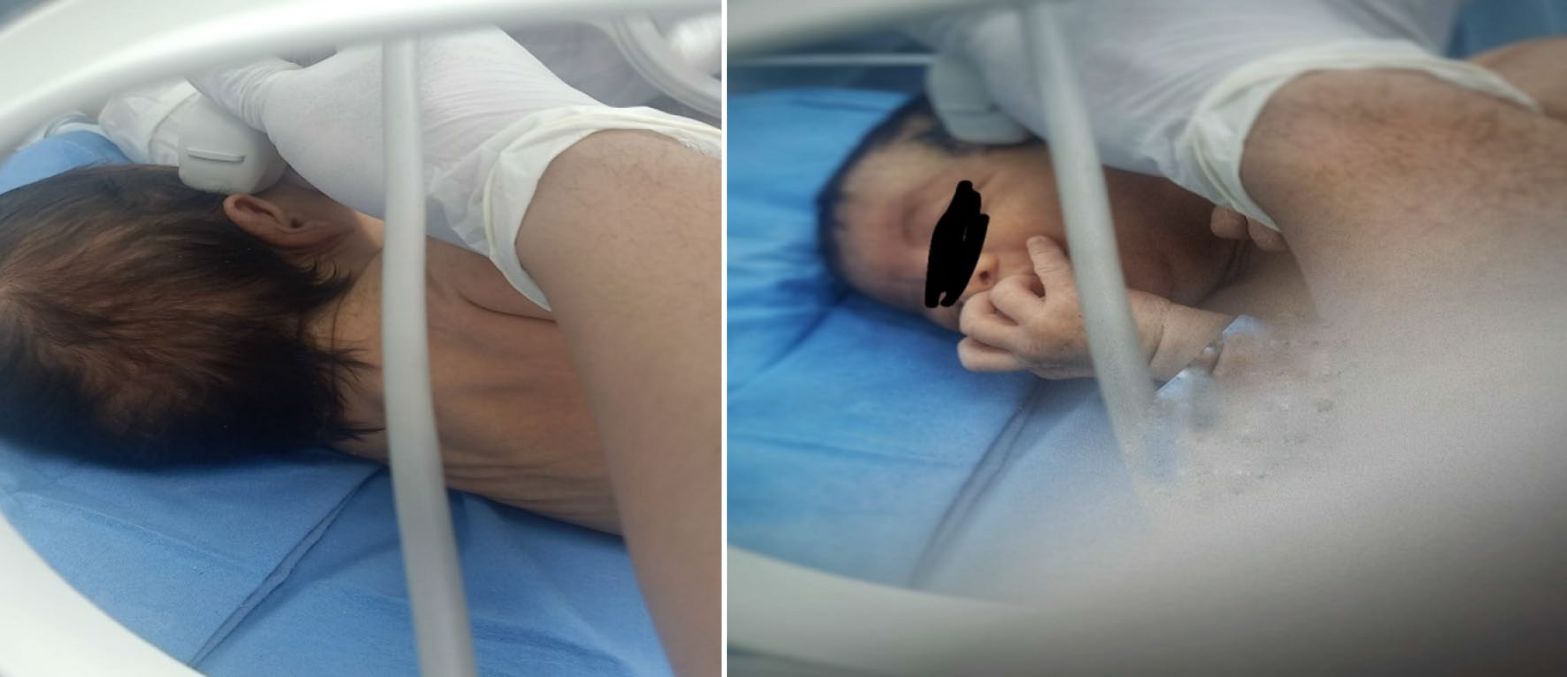
Trans-temporal approach with lateral tilting to either side

The patient lies supine with lateral tilting of his head to either side during examination (Fig. 1). The two middle cerebral, internal carotid and posterior cerebral arteries were examined using trans-temporal approach. The probe was placed on the temporal aspect of the head, cephalad to the zygomatic arch and immediately anterior and superior to the tragus of the ear in a transverse position.

The beam was angulated anteriorly. The anterior orientation of the beam allowed for the investigation of the M1 and M2 segments of the middle cerebral arteries (MCAs).When the midbrain is observed, color Doppler was applied and the circle of Willis became evident. The middle cerebral artery was directed laterally into sylvian fissure.

Then spectral wave analysis was done in M1 segment on both sided with angle around 60° and sample volume 2.5 mm.

- After angling the probe slightly inferiorly and posteriorly, the distal part of internal carotid arteries was visualized, with color flow in direction to the transducer.
- The transducer often needs to be slid posteriorly for the posterior cerebral arteries, with color flow was directed in direction to the transducer in its initial part then away from the transducer as it courses around the cerebral peduncle.

The following parameters were measured on both sides (right and left sides): peak systolic velocity (PSV),resistivity index (RI) and pulsatility index (PI).

PI which is the difference between the peak systolic flow and minimum diastolic flow velocity, divided by the mean velocity. PI = (VS VD) / TAMAX while, RI = (VS – VD)/VS.TAMAX is time average mean of maximal velocities, often known as mean velocity. (VS) peak systolic velocity and (VD)end diastolic velocity.

### Statistical analysis

Data were coded and entered using the statistical package for the Social Sciences (SPSS) version 28 (IBM Corp., Armonk, NY, USA). Data were summarized using mean and standard deviation for quantitative variables. Frequencies (number of cases) and relative frequencies (percentages) for categorical variables. Comparisons between groups were done using unpaired *t*-test. For comparing categorical data, Chi square (*χ*^2^) test was performed. Exact test was used instead when the expected frequency is less than 5. Correlations between quantitative variables were done using Pearson correlation coefficient. *p*-values less than 0.05 were considered as statistically significant.

Correlation of transcranial Doppler parameters with total serum bilirubin level, acute bilirubin encephalopathy (according to the BIND score) and the different lines of management, was done.

## Results

In this prospective observational case control study, 26 neonates with extreme unconjugated hyper-bilirubinemia (group A) were compared with 13 full term age and sex matched normal healthy neonates (group B) serving as control. The mean age of the case group was 4.50 ± 1.68 days for group A and 4.46 ± 1.05 days for group B. Males were 61.5% and 69.2% for group A and group B respectively. Demographic data are comparable between the two groups demonstrating homogeneity of the study population (Table 1).

**Table 1:** Demographic data of the studied population

	(Group A) *n* = 26	(Group B) *n* = 13	*p* value
Post-natal age (days)	4.50 ± 1.68	4.46 ± 1.05	0.940
Sex			
Female	10 (38.5%)	4 (30.8%)	0.733
Male	16 (61.5%)	9 (69.2%)	
Mode of delivery			
NVD	8 (30.8%)	5 (38.5%)	0.733
C.S	18 (69.2%)	8 (61.5%)	

T-test

Chi square test

*NVD*: normal vaginal delivery. *C.S*: Cesarean section

As for the clinical data, 69.2% of the patients had no neurological dysfunction, while the rest had different degrees of neurological dysfunction. Half of the patients were treated by exchanges transfusion and the other half by intensive phototherapy (Table 2).

**Table 2:** Clinical and laboratory data of the neonates with unconjugated hyper-bilirubinemia (group A)

	Count	%
ABE		
Normal (BIND 0)	18	69.2%
Mild BIND (1–3)	7	26.9%
Moderate BIND (4–7)	1	3.9%
Severe BIND (> 7)	0	0%
Management		
Exchange transfusion	13	50.0%
Intensive phototherapy	13	50.0%
	Mean	Standard Deviation
Laboratory data		
Total serum (mg/dl) bilirubin level	22.81	± 1.77
Direct serum bilirubin level (mg/dl)	0.67	± 0.20

*BIND:* bilirubin induced neurological dysfunction, *ABE*: acute bilirubin encephalopathy, *Mg/dl*: Milligrams per deciliter

Regarding the measured color Doppler parameters, the resistance index (RI) of the right and left posterior cerebral arteries are lower in group A versus group B (*p* values: < 0.001 and 0.019 respectively). The pulsatility index (PI) of the left middle cerebral and right internal carotid arteries are lower in group A versus group B (*p* values: 0.011 and 0.002, respectively). The pulsatility index (PI) of the right and left posterior cerebral arteries are lower in UCH cases group A versus group B (P values: 0.012 and 0.008, respectively) (Table 3).

**Table 3:** Transcranial Doppler parameters of the studied population

			*p* value
	Group A	Group B	
	Mean ± SD	Mean ± SD	
Peak systolic velocity (PSV) (cm/s)			
PSV RT MCA	120.38 ± 25.92	107.08 ± 13.48	0.092
PSV LT MCA	121.08 ± 25.63	110.54 ± 17.45	0.191
PSV RT ICA	101.38 ± 16.43	99.00 ± 13.71	0.655
PSV LT ICA	105.08 ± 18.37	99.62 ± 16.74	0.374
PSV RT PCA	79.38 ± 19.00	74.23 ± 24.11	0.470
PSV LT PCA	80.62 ± 19.33	81.54 ± 23.69	0.897
Resistivity indices (RI and PI)			
RI RT MCA	0.65 ± 0.09	0.68 ± 0.11	0.263
RI LT MCA	0.66 ± 0.07	0.70 ± 0.10	0.107
RI RT ICA	0.65 ± 0.08	0.69 ± 0.10	0.161
RI LT ICA	0.65 ± 0.06	0.71 ± 0.10	0.073
RI RT PCA	0.60 ± 0.09	0.74 ± 0.11	< 0.001*
RI LT PCA	0.60 ± 0.09	0.68 ± 0.11	0.019*
PI RT MCA	1.01 ± 0.16	1.23 ± 0.35	0.057
PI LT MCA	1.06 ± 0.19	1.33 ± 0.31	0.011*
PI RT ICA	1.06 ± 0.18	1.30 ± 0.26	0.002*
PI LT ICA	1.01 ± 0.17	1.23 ± 0.38	0.065
PI RT PCA	0.94 ± 0.20	1.18 ± 0.36	0.012*
PI LT PCA	0.93 ± 0.18	1.17 ± 0.37	0.008*

*PSV* peak systolic velocity, *RT* right, *MCA* middle cerebral artery, *LT* left, *ICA* internal carotid artery, *PCA* posterior cerebral artery, *RI* resistance index, *PI* pulsatility index, *cm/s* centimeters per second

*p*-values less than 0.05 were considered as statistically significant

The bold values to show the titles and to highlight the significant values

**Fig. 2:**
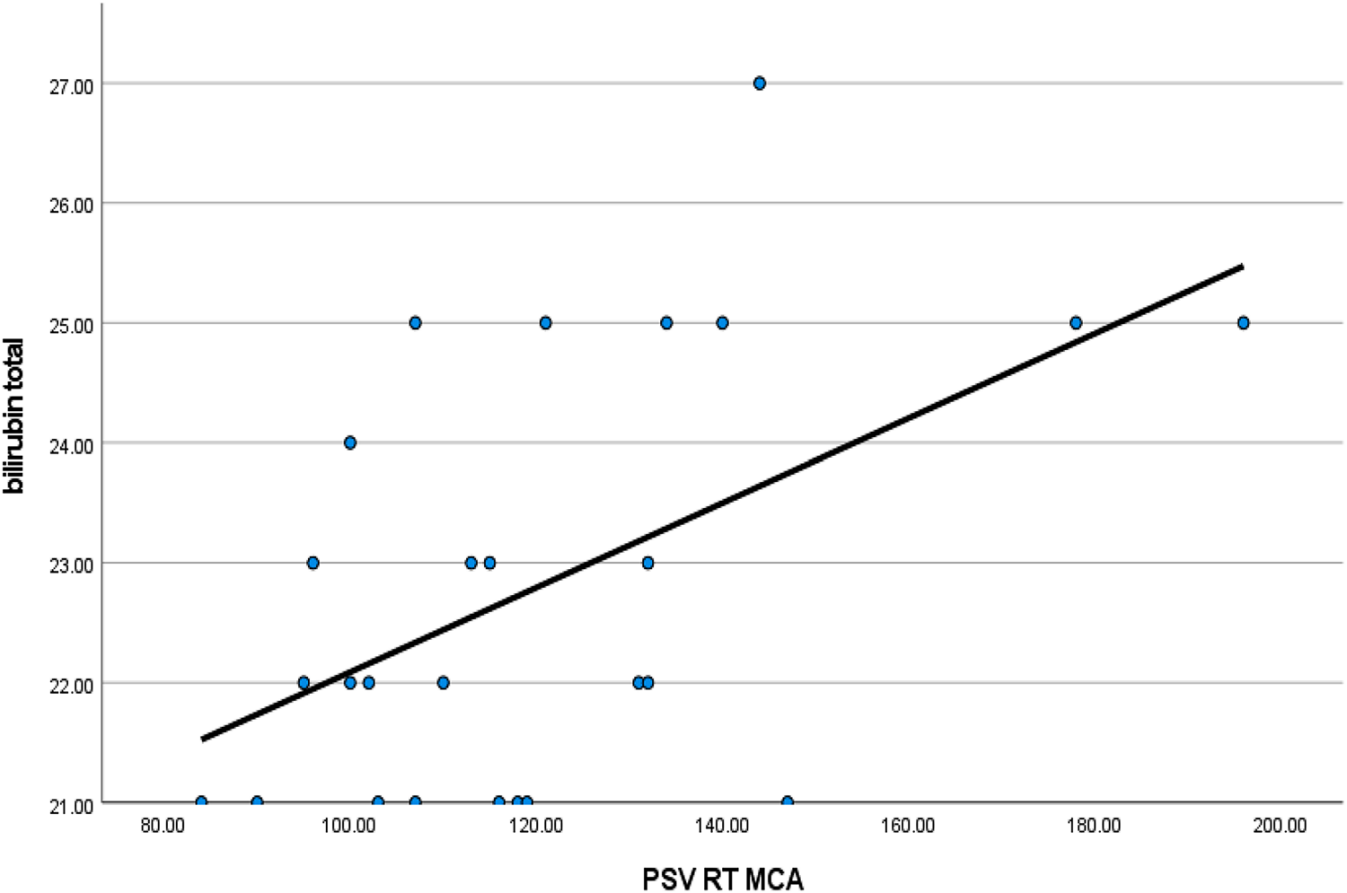
Positive correlation between serum total bilirubin level and peak systolic velocity in the right middle cerebral artery

When assessing the serum total bilirubin and correlating it to the different Doppler parameters, we found a strong positive correlation between serum total bilirubin level and peak systolic velocity in the right middle cerebral artery (*r* = 0.517, *p* = 0.007*).The higher serum total bilirubin level, the higher peak systolic velocity due to increased cerebral blood flow in babies with extreme conjugated hyperbilirubinemia (Table 4) (Fig. 2).

**Table 4:** Correlation of transcranial Doppler parameters with total serum bilirubin level

	Total serum bilirubin level (mg/dL)		
	Pearson correlation	Sig. (2-tailed)	*N*
Peak systolic velocity (PSV)			
PSV RT MCA	**0.517**	0.007*	26
PSV LT MCA	0.385	0.052	26
PSV RT ICA	0.379	0.056	26
PSV LT ICA	0.026	0.898	26
PSV RT PCA	0.170	0.405	26
PSV LT PCA	0.105	0.608	26
Resistivity indices (RI and PI)			
RI RT MCA	**– 0.435**	0.026*	26
RI LT MCA	**– 0.409**	0.038*	26
RI RT ICA	– 0.117	0.568	26
RI LT ICA	– 0.255	0.208	26
RI RT PCA	– 0.136	0.507	26
RI LT PCA	**– 0.434**	0.027*	26
PI RT MCA	**– 0.486**	0.012*	26
PI LT MCA	– 0.156	0.445	26
PI RT ICA	– 0.253	0.212	26
PI LT ICA	– 0.185	0.365	26
PI RT PCA	– 0.286	0.157	26
PI LT PCA	– 0.249	0.220	26

*Mg/dL* milligrams per deciliter, *PSV* peak systolic velocity, *RT* right, *MCA* middle cerebral artery, *LT* left, *ICA* internal carotid artery, *PCA* posterior cerebral artery, *RI* resistance index, *PI* pulsatility index

*p*-values less than 0.05 were considered as statistically significant

The bold values to show the titles and to highlight the significant values

**Fig. 3:**
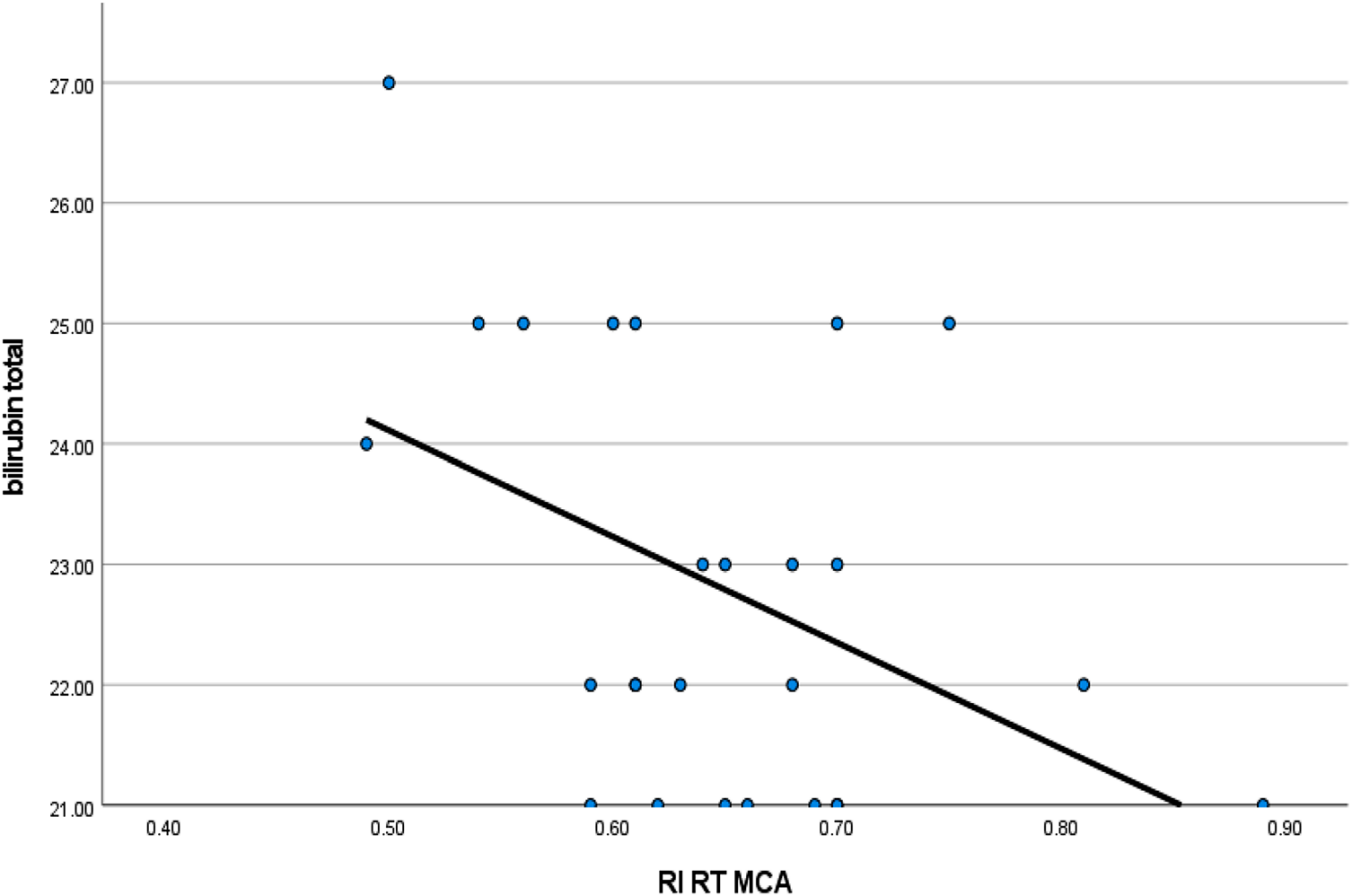
Negative correlation between serum total bilirubin level and resistance index in the right middle cerebral artery

There is a strong negative correlation between serum total bilirubin level and resistivity index in the right middle cerebral artery (*r* = − 0.435, *p* = 0.026),left middle cerebral artery (*r*  = -– 0.409, p = 0.038) and left posterior cerebral artery (*r* = − 0.434, *p* = 0.027). The higher serum total bilirubin level, the lower is resistivity index (RI) due to increased cerebral blood flow in babies with extreme conjugated hyperbilirubinemia (Fig. 3).

**Fig. 4.**
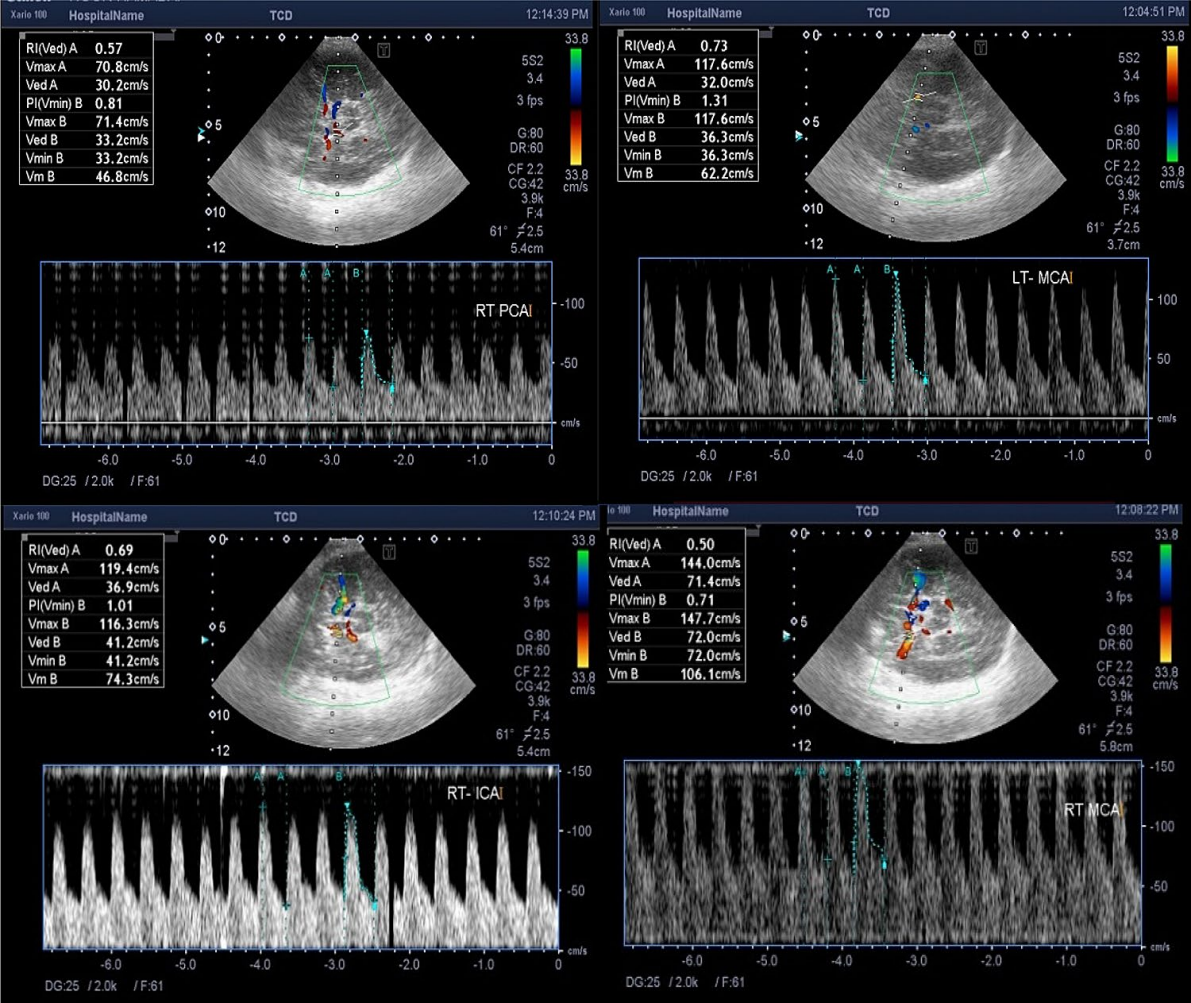
7 days old full term female neonate presented with jaundice. Trans-cranial Doppler measurement showing reduced RI and PI of right MCA and PCA

**Fig. 5.**
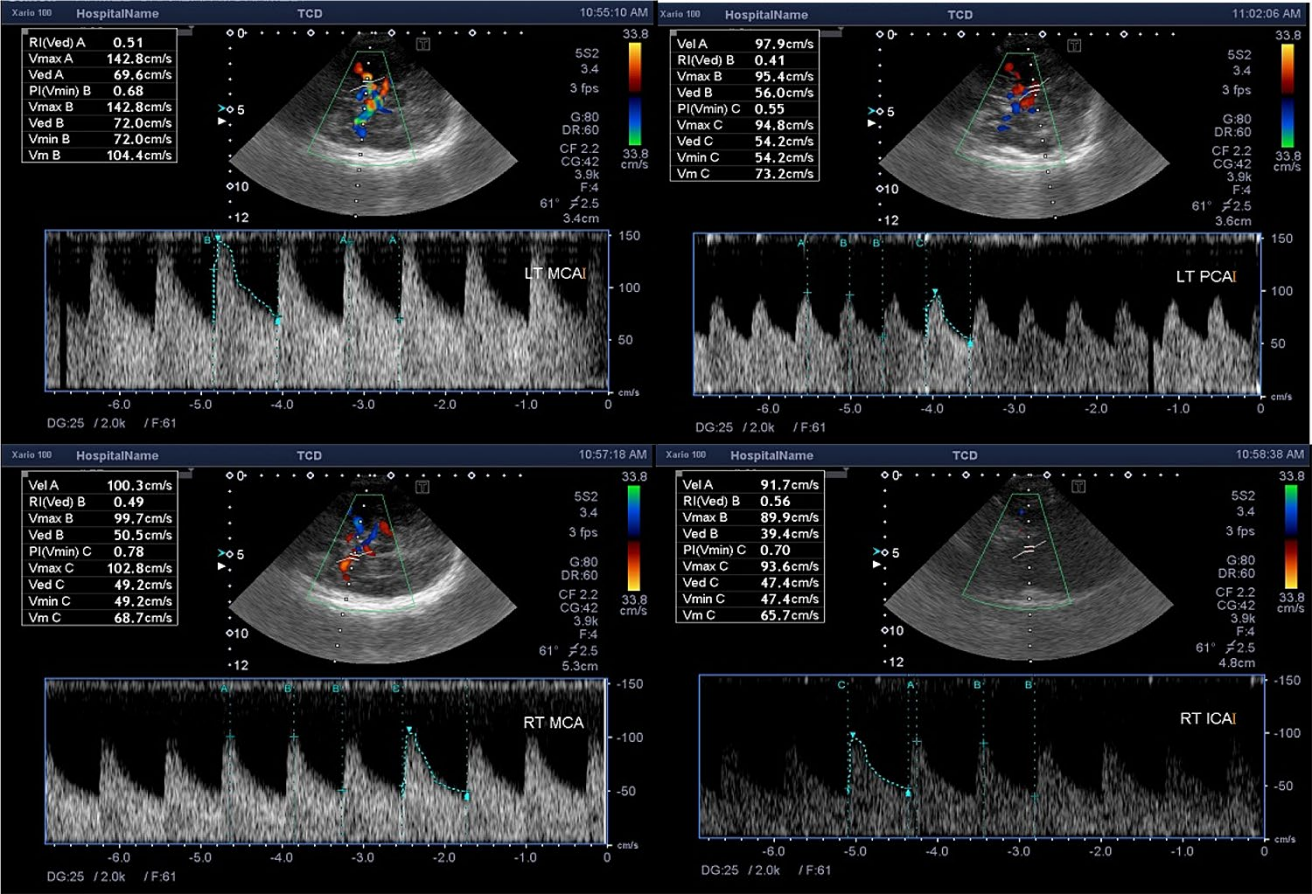
2 days old full term male neonate presented with jaundice. Trans-cranial Doppler measurement showing reduced RI and PI of both MCAs, left PCA and right ICA with elevated PSV of left MCA

**Fig. 6.**
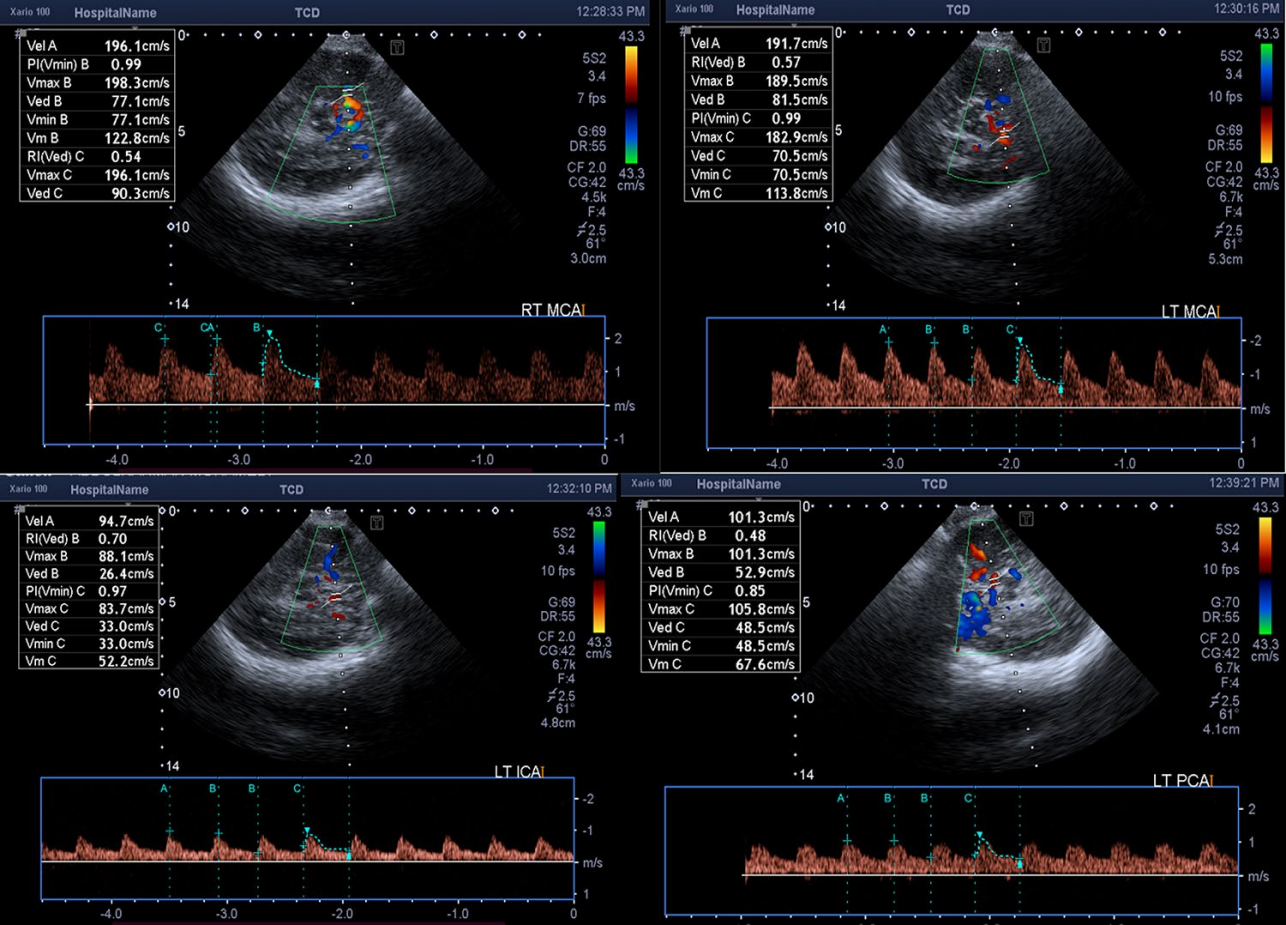
5 days old full term male neonate presented with jaundice. Trans-cranial Doppler measurement showing increased PSV and reduced RI and PI of both MCAs with reduced RI and PI of the left PCA

There is a strong negative correlation between serum total bilirubin level and pulsatility index in the right middle cerebral artery (*r* = − 0.486, *p* = 0.012). The higher serum total bilirubin level, the lower is the pulsatility index (RI) due to increased cerebral blood flow in babies with extreme conjugated hyperbilirubinemia. Examples for changes in different Color Doppler parameters, in our cases is shown in (Figs. 4, 5 and 6).

On correlating the Doppler measures for the different intracranial arteries and the acute bilirubin encephalopathy (as determined by the BIND score), we found significant positive correlation between peak systolic velocity and acute bilirubin encephalopathy in the right middle cerebral artery (*p*-value: 0.037). Peak systolic velocity is higher in cases with ABE reflecting increased cerebral blood flow (Table 5).

**Table 5:** Correlation of transcranial Doppler parameters with acute bilirubin encephalopathy

	ABE		No ABE		
	Mean	Standard deviation	Mean	Standard deviation	*p* value
Peak systolic velocity (PSV)					
PSV RT MCA	154.60	31.33	112.24	16.76	0.037*
PSV LT MCA	144.80	35.74	115.43	19.79	0.141
PSV RT ICA	108.80	17.48	99.62	16.10	0.270
PSV LT ICA	102.20	11.95	105.76	19.76	0.705
PSV RT PCA	90.00	28.20	76.86	16.04	0.365
PSV LT PCA	86.80	12.62	79.14	20.58	0.437
Resistivity indices (RI and PI)					
RI RT MCA	0.60	0.10	0.66	0.08	0.176
RI LT MCA	0.64	0.08	0.66	0.07	0.472
RI RT ICA	0.66	0.11	0.65	0.07	0.701
RI LT ICA	0.63	0.06	0.66	0.07	0.396
RI RT PCA	0.58	0.13	0.61	0.08	0.577
RI LT PCA	0.55	0.09	0.61	0.08	0.124
PI RT MCA	0.95	0.18	1.03	0.16	0.316
PI LT MCA	1.09	0.15	1.05	0.20	0.692
PI RT ICA	1.02	0.10	1.06	0.20	0.662
PI LT ICA	0.97	0.12	1.02	0.18	0.547
PI RT PCA	0.91	0.15	0.95	0.22	0.693
PI LT PCA	0.94	0.19	0.93	0.18	0.905

*PSV* peak systolic velocity, *RT* right, *MCA* middle cerebral artery, *LT* left, *ICA* internal carotid artery, *PCA* posterior cerebral artery, *PI* pulsatility index, *RI* resistance index, *ABE* acute bilirubin encephalopathy

*p*-values less than 0.05 were considered as statistically significant

The plan of management,whether exchanges transfusion or intensive phototherapy,was correlated with the PSV,RI and the PI. We found that the resistance indices (RI) of the right middle cerebral artery and left posterior cerebral artery are significantly lower in patients undergone exchange transfusion (*p*-values: 0.043 and 0.008 respectively). The pulsatility indices of the right internal cerebral artery, right and left posterior cerebral arteries are significantly lower in patients undergone exchange transfusion (*p*-values: 0.025, 0.011 and 0.046 respectively) (Table 6).

**Table 6:** Correlation of the transcranial Doppler parameters with lines of management

	Exchange transfusion		Intensive phototherapy		
	Mean	Standard deviation	Mean	Standard deviation	*p* value
Peak systolic velocity (PSV)					
PSV RT MCA	129.23	30.27	111.54	17.71	0.081
PSV LT MCA	130.31	27.11	111.85	21.20	0.065
PSV RT ICA	103.77	19.82	99.00	12.53	0.471
PSV LT ICA	103.23	14.96	106.92	21.71	0.618
PSV RT PSA	85.38	21.04	73.38	15.20	0.109
PSV LT PSA	83.46	17.68	77.77	21.18	0.464
Resistivity indices (RI and PI)					
RI RT MCA	0.61	0.08	0.68	0.08	0.043*
RI LT MCA	0.63	0.07	0.68	0.07	0.059
RI RT ICA	0.63	0.08	0.67	0.07	0.227
RI LT ICA	0.64	0.07	0.66	0.06	0.453
RI RT PCA	0.60	0.10	0.61	0.08	0.618
RI LT PCA	0.56	0.08	0.64	0.08	0.008*
PI RT MCA	0.96	0.15	1.06	0.17	0.120
PI LT MCA	1.00	0.20	1.12	0.17	0.110
PI RT ICA	0.98	0.15	1.14	0.18	0.025*
PI LT ICA	0.97	0.17	1.04	0.17	0.322
PI RT PCA	0.84	0.16	1.04	0.20	0.011*
PI LT PCA	0.86	0.15	1.00	0.18	0.046*

*PSV* peak systolic velocity, *RT* right, *MCA* middle cerebral artery, *LT* left, *ICA* internal carotid artery, *PCA* posterior cerebral artery, *PI* pulsatility index, *RI* resistance index

*p*-values less than 0.05 were considered as statistically significant

## Discussion

Hyperbilirubinemia is a disorder in which bilirubin levels in the blood are elevated, resulting in a yellowish staining of the eyes and skin. In the first week of life, 50–60 percent of newborns have hyperbilirubinemia [12]**.** Accelerated bilirubin production is caused by increased breakdown of erythrocytes harbouring foetal Hgb, inadequate erythropoiesis, reduced conjugation due to liver enzyme immaturity, and a unique neonatal phenomena of enterohepatic bilirubin recirculation during the first week of life, contributing to newborn jaundice [13].

This prospective cross sectional study was conducted on 26 full term neonates with extreme unconjugated hyperbilirubinemia,admitted at the neonatal intensive care unit and fulfilling the inclusion criteria, aiming to assess the peak systolic velocity (PSV), Resistivity index (RI) and pulsatility index (PI) among patients with extreme neonatal jaundice. They were compared to 13other age and sex matched full term neonates with normal bilirubin level and admitted for surgical intensive care unit.

In the present study, the highest mean peak systolic velocity recorded among our unconjugated hyper-bilirubinemia patients was 121.08 ± 25.63 cm/s. It was noted in the left MCA compared to 110.54 ± 17.45 in the control group. On the other hand the least mean velocity was 110.54 ± 17.45 and noted at the right PCA compared to 74.23 ± 24.11 at the control group. The lowest mean resistivity index was 0.60 ± 0.09 noted at the right and left PCAs compared to 0.74 ± 0.11 and 0.68 ± 0.11, respectively in the control group. While the lowest mean pulsatility index was 0.93 ± 0.18 noted at the left PCA compared to 1.17 ± 0.37 in the control group. These Doppler findings are consistent with the study conducted by Basu et al.[14], who stated that there is an increase in the cerebral blood flow, evidenced by increased peak systolic velocity and decreased resistivity indices in neonates with extreme unconjugated hyperbilirubinemia compared to healthy ones.

We also found that serum level of total bilirubin was positively correlated with peak systolic velocity in the right middle cerebral artery. This is in concordance to one study performed by Salama et al. [15],who correlated hemolytic markers with TCD velocities and found that increased cerebral blood velocities were positively correlated with elevated total serum bilirubin levels. It was also inversely correlated with each of the resistance and pulsatiliy indices of the same artery. Moreover, it was also found to be inversely correlated with the resistivity index in left middle cerebral artery, resistivity index in left posterior cerebral artery and pulsatility index in right middle cerebral artery.

An increase in cerebral blood flow velocity (CBFV) was also reported by Basu et al. [14] study, in terms of decreased resistance, increased blood flow and vasodilation in neonates with significant non-hemolytic UCH, when compared with those without icterus.

When evaluating the patients neurologically, we found that according to BIND score described by Gamaleldin et al. [16], 18 patients (69.2%) did not show any features of neurological dysfunction i.e. bilirubin encephalopathy, while 7 (26.9%) patients developed mild clinical features of neonatal ABE (BIND score 1–3) and one patient (3.9%) was developing moderate ABE clinical features (BIND 4–7). These percentages are close to the relative values found by Smitherman et al. [17],who studied the incidence of ABE and correlated it with hyperbilirubinemia. They found that the incidence of ABE is about 1–2% in patients with total serum bilirubin level more than 20 md/dL.

On correlating the peak systolic velocity in the targeted intracranial arteries with the BIND score and the severity of ABE, we found that there is a positive correlation between peak systolic velocity in the right middle cerebral artery and the ABE. This is consistent with Basu et al. [14]. It is well known that the development of ABE is determined by gestation, birth weight, associated morbidity like sepsis and gastrointestinal obstruction, peak of total serum bilirubin (TSB), age at peak TSB, serum albumin, and the presence of hemolysis. But unlike the study conducted by Basu et al. [14], In our study we excluded all possible variables like perinatal asphyxia, shock, sepsis, anemia, systemic and metabolic disorders, hypo/ hyperthermia and inter-observer variation which could have influenced the Color Doppler parameters, yet we got the same results.

In our study, 13 (50%) UCH patients were managed by intensive phototherapy, while 13 patients (50%) were managed by exchange transfusion. It was found that the resistance index of the right middle cerebral and left posterior cerebral arteries are significantly lower in patients managed by exchange transfusion compared to patients managed by intensive phototherapy. Moreover, the pulsatility index of the right internal carotid artery and both posterior cerebral arteries are also significantly lower in patients managed by exchange transfusion. The aforementioned findings are likely reflecting increased cerebral blood flow in those patients with risk of brain affection in patients managed by exchange transfusion.

The major limitations of this study were that we could not conduct a follow up study after treatment whether by intensive phototherapy or exchange transfusion for a more accurate interpretation of the results as we couldn’t retain or get back the patients for research purposes, thus the interpretation of the statistical validity tests may not be totally accurate. Moreover long-term developmental outcome was not evaluated and no other radiological investigations were performed, such as an MRI (being expensive with the need for sedation).

## Conclusions

To conclude, this study documents an increase in cerebral blood flow velocity in neonates with unconjugated hyperbilirubinemia compared with those without it, it also states significant correlation between cerebral blood flow velocity and bilirubin level. Though transcranial Doppler parameters have shown changes in neonates with acute bilirubin encephalopathy, its predictive accuracy is questionable considering the small number of events. A larger series may be considered to study the statistical validity of cerebral blood flow velocity as an early predictor of impending neuronal damage in neonatal unconjugated hyperbilirubinemia.

## Data Availability

The data supporting the conclusions of this article are available upon reasonable request from the authors.

## Abbreviations

ABE: Acute bilirubin encephalopathy
BIND: Bilirubin induced neurological dysfunction
C.S: Cesarean section
ICA: Internal carotid artery
MCA: Middle cerebral artery
NVD: Normal vaginal delivery
PCA: Posterior cerebral artery
PI: Pulsatility index
PSV: Peak systolic velocity
RI: Resistance index
UCH: Unconjugated hyperbilirubinemia
